# Supplementary material for: Near real-time enumeration of live and dead bacteria using a fibre-based spectroscopic device
Source: Sci Rep. 2019 Mar 18;9:4807. doi: 10.1038/s41598-019-41221-1 (PMC6423134; doi:10.1038/s41598-019-41221-1)
Supplement: Supplementary file 1 — Supplementary information [file 41598_2019_41221_MOESM1_ESM.pdf]

Supplementary information for: Near real-time enumeration of live and dead bacteria using a fibre-based spectroscopic device

Authors: Fang Ou[1,2], Cushla McGoverin[1,2], Simon Swift[3], Frédérique Vanholsbeeck[1,2]

[1] Department of Physics, The University of Auckland, Auckland, New Zealand

[2] The Dodd-Walls Centre for Photonic and Quantum Technologies, New Zealand

[3] School of Medical Sciences, The University of Auckland, Auckland, New Zealand

Sample type	Experiment number	Mean concentration of live cell suspension (bacteria/mL)	Mean concentration of dead cell suspension (bacteria/mL)
Training samples	1	$(6.50 \pm 0.18) \times 10^7$	$(4.38 \pm 0.03) \times 10^7$
	2	$(5.56 \pm 0.01) \times 10^7$	$(3.45 \pm 0.00) \times 10^7$
	3	$(1.36 \pm 0.01) \times 10^8$	$(1.08 \pm 0.02) \times 10^8$
	4	$(9.42 \pm 0.12) \times 10^7$	$(4.32 \pm 0.01) \times 10^7$
	5	$(9.47 \pm 0.09) \times 10^6$	$(7.53 \pm 0.02) \times 10^6$
	6	$(1.14 \pm 0.01) \times 10^7$	$(5.20 \pm 0.01) \times 10^6$
	7	$(9.32 \pm 0.05) \times 10^6$	$(7.32 \pm 0.03) \times 10^6$
Test set samples	8	$(1.17 \pm 0.13) \times 10^8$	$(1.17 \pm 0.13) \times 10^8$
	9	$(9.05 \pm 0.39) \times 10^7$	$(9.05 \pm 0.39) \times 10^7$

Supplementary Table 1. The mean and standard error of the concentration of live and dead cell suspensions that were used to make bacterial mixtures with varying proportions of live and dead cells, measured by flow cytometry (FCM). For the training samples, the concentration of live and dead suspensions were obtained from triplicate FCM measurements of the 100% and 0% live samples, respectively. The mean concentration of live cell suspensions of test set samples were calculated from the FCM-measured live cell concentration and FCM-measured percentage of live cells the final bacterial mixtures. Vice versa for the mean concentration of dead cell suspensions of test set samples.

Sample type	Experiment number	Volume of live cell suspension (µL)	Volume of dead cell suspension (µL)	Volume of saline diluent (µL)	Expected % live in the final mixture
Training samples	1 - 7	0	3000	0	0
		75	2925	0	2.5
		150	2850	0	5
		300	2700	0	10
		750	2250	0	25
		1500	1500	0	50
		2250	750	0	75
		3000	0	0	100
Test set samples	8	45	255	2700	15
		105	195	2700	35
		165	135	2700	55
		210	90	2700	70
		285	15	2700	95
		750	2250	0	25
		1350	1650	0	45
		1050	1950	0	35
	9	84	1116	0	7
		252	948	0	21
		840	360	0	70
		432	768	0	36
		672	528	0	56
		540	660	0	45
		936	264	0	78
		168	1032	0	14
		4.8	115.2	1080	4
		21.6	98.4	1080	18
		34.8	85.2	1080	29
		45.6	74.4	1080	38
		82.8	37.2	1080	69
		66	54	1080	55
		92.4	27.6	1080	77
		51.6	68.4	1080	43
		35	11.7	1153.3	75
		26.6	20.1	1153.3	57
		5.6	41.1	1153.3	12

Supplementary Table 2. The volumes of live and dead cell suspensions and saline diluent combined to make bacterial mixtures that contain varying proportions of live and dead cells.
